# Chromium carbide micro-whiskers dataset: Morphologies with scanning and transmission electronic microscopy

**DOI:** 10.1016/j.dib.2020.106222

**Published:** 2020-08-24

**Authors:** Chenxi Zhai, Mingchao Wang, Zhaojie Feng, Qingjun Zhou, Tong Wei, Jiachen Liu

**Affiliations:** a Sibley School of Mechanical and Aerospace Engineering, Cornell University, Ithaca, NY, 14853, USA; b Department of Mechanical Engineering, FAMU-FSU College of Engineering, Florida State University, Tallahassee, FL, 32310, USA; c College of Science, Civil Aviation University of China, Tianjin 300300, PR China; d School of Materials Science and Engineering, Tianjin University, Tianjin 300072, PR China

**Keywords:** Chromium carbide, Whiskers, Growth mechanism, Strengthening

## Abstract

Chromium carbide exhibits a superior set of mechanical properties and chemical stabilities and is widely used in various engineering applications. Here, micro-whiskers of the Cr_2_O_3_ were successfully prepared using a carbothermal reduction method with high energy milling and liquid phase catalysis. The whiskers growth was observed with scanning electron microscopy and field emission gun transmission electron microscopy. This dataset shows all kinds of morphologies of the Cr_2_O_3_ whiskers during the growth stage, including agglomerated, pointed, and non-whiskered shapes, which are products of the mixture of Cr_2_O_3_:C = 1:5 or 1:8 or 1:10 under different temperatures and duration time. These data provide important additional information different from the source article but complement it with some negative but indicative and instructive data. Experimental scientists who want to investigate the growth and strengthening of whiskers of Cr_2_O_3_ or others can refer to and benefit from these data, such as possible combinations of the experimental conditions which may lead to certain outcomes and guide the predictive design of future relevant research with similar materials system.

**Specifications Table**

**Table:** 

Subject	Ceramics and Composites
Specific subject area	Whiskers, strengthening, crystal growth
Type of data	Image
How data were acquired	The data of morphologies of the chromium carbide whiskers were observed with SEM (scanning electron microscopy), S-4800, Hitachi, Tokyo, Japan and crystal structure and composition analysis were characterized by field emission gun TEM (transmission electron microscopy), Tecnai G2 F20, FEI, Eindhoven, Netherlands with an EDS (energy dispersive X-ray detector).
Data format	Raw
Parameters for data collection	stoichiometric ratio, temperature, time
Description of data collection	these data were collected by SEM and TEM during the whisker growth under various experimental conditions
Data source location	Institution: City/Town/Region: Tianjin Country: China
Data accessibility	With the article
Related research article	M. Wang, Z. Feng, C. Zhai, Q. Zhou, T. Wei, J. Liu, Chromium carbide micro-whiskers: preparation and strengthening effects in extreme conditions with experiments and molecular dynamics simulations, J. Solid State Chem. 291 (2020) 121598 doi: 10.1016/j.jssc.2020.121598

## Value of the Data

• These data provide important additional information which is different from the source articles but can successfully complement this systematic approach, with some negative but indicative and instructive data.
• Experimental scientists who want to investigate the growth and strengthening of whiskers of chromium carbide or others can refer to and benefit from these data.
• These data can be re-used as a guidance with specific experimental conditions to generate chromium-carbide whiskers with morphologies shown in the SEM/TEM data.
• The SEM and TEM data clearly showed some possible combinations of the experimental conditions which may lead to certain outcomes and will guide the predictive design of future relevant research with similar materials system.

## 1 Data Description

**Fig. 1:**
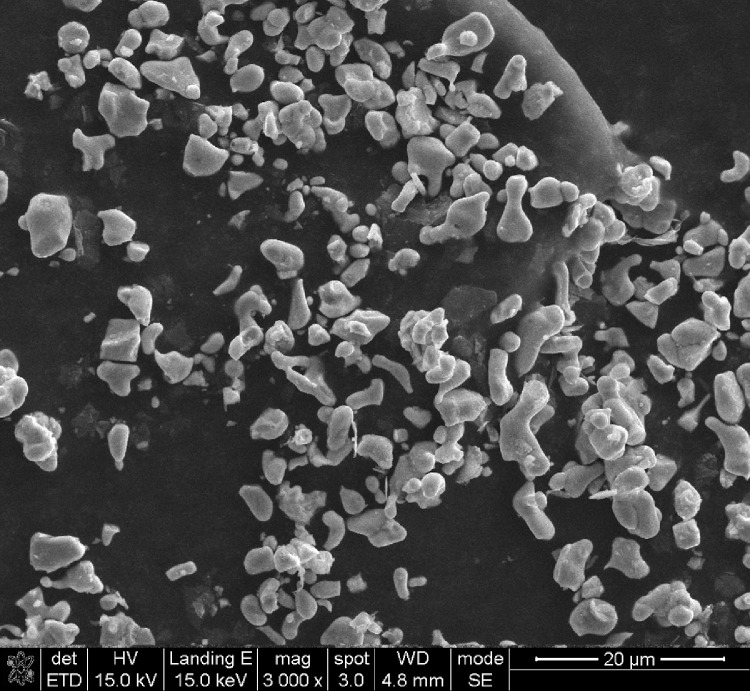
SEM images of products of mixture of Cr2O3:C = 1:5 under conditions of 1300°C - 1 h.

**Fig. 2:**
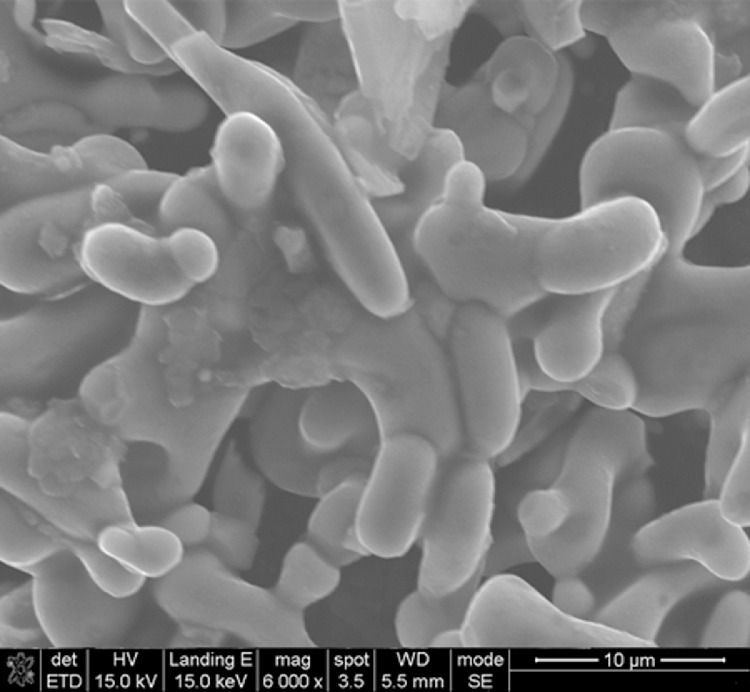
SEM images of products of mixture of Cr2O3:C = 1:5 under conditions of 1300°C-4 h.

**Fig. 3:**
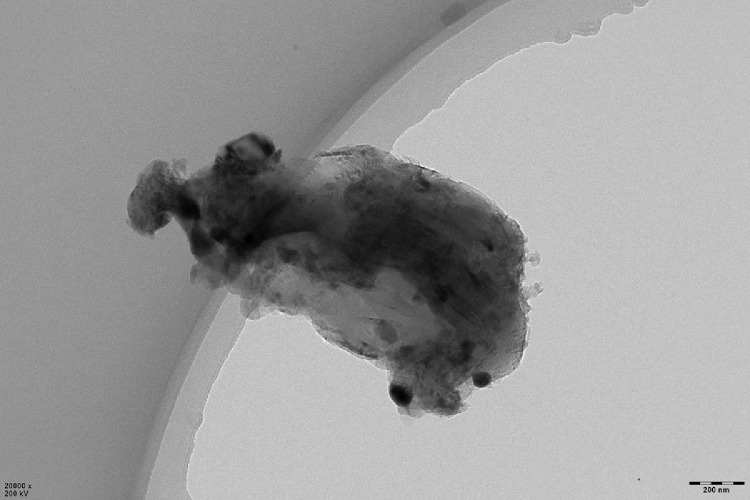
TEM images of non-whiskers products of mixture of Cr2O3:C = 1:5 under conditions of 1300°C - 4 h.

**Fig. 4:**
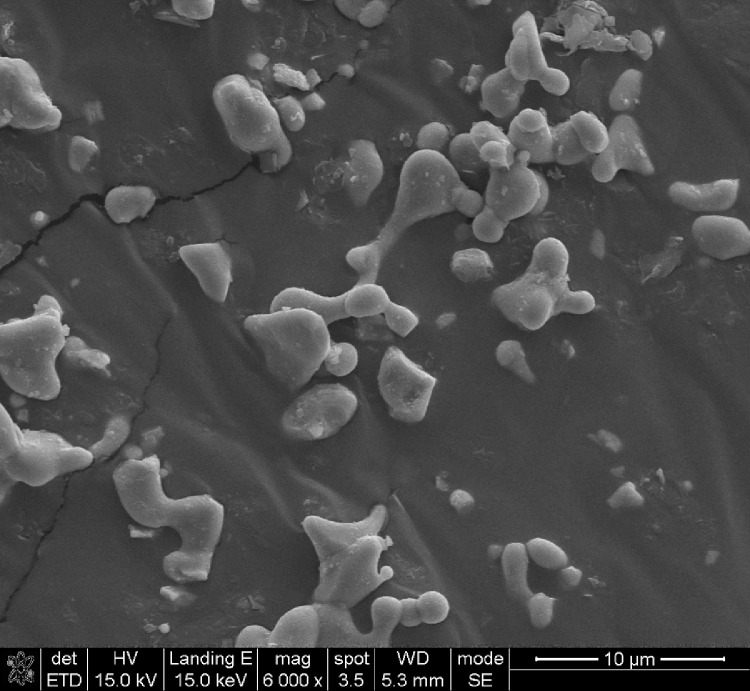
SEM images of products of mixture of Cr2O3:C = 1:10 under conditions of 1300°C - 1 h.

**Fig. 5:**
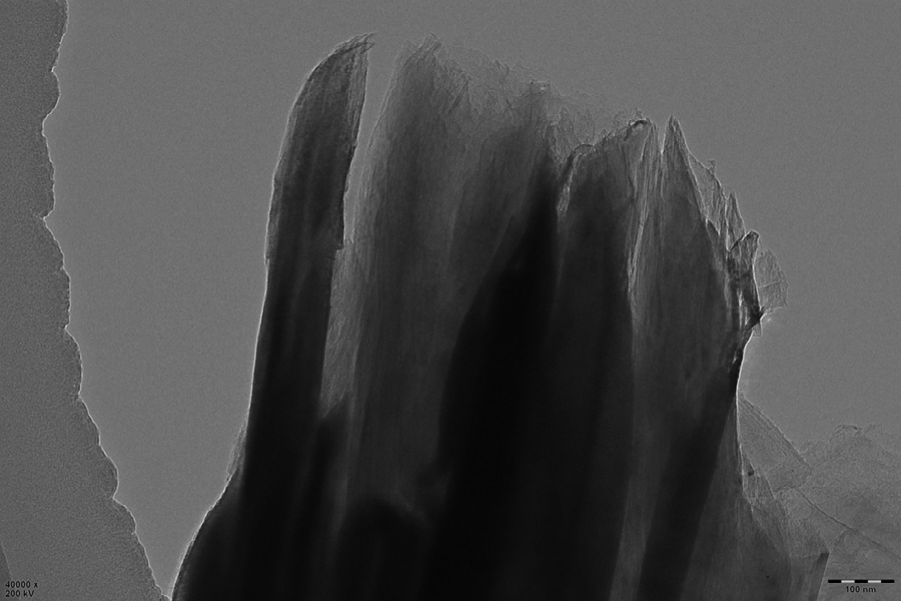
TEM images of products with agglomerated structures of mixture of Cr2O3:C = 1:10 under conditions of 1300°C - 2 h.

**Fig. 6:**
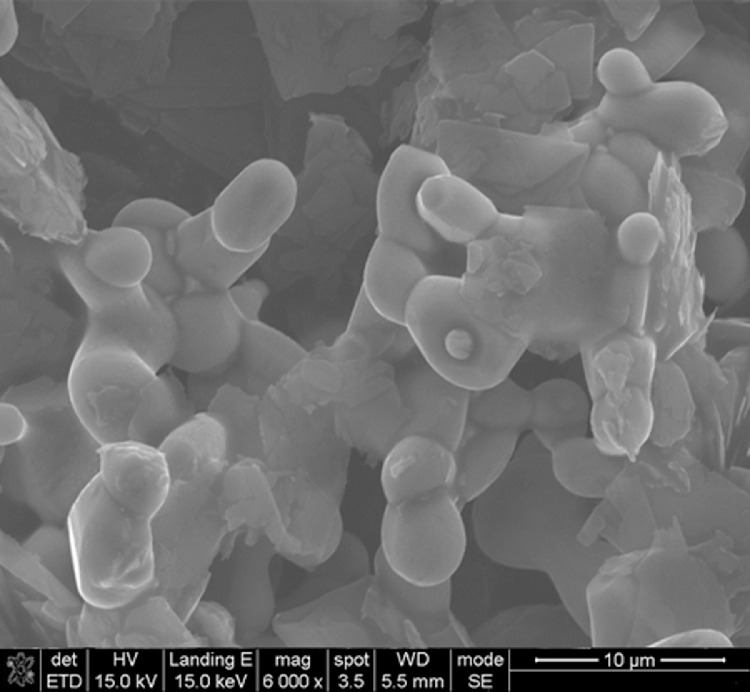
SEM images of products of mixture of Cr2O3:C = 1:10 under conditions of 1300°C - 4 h.

**Fig. 7:**
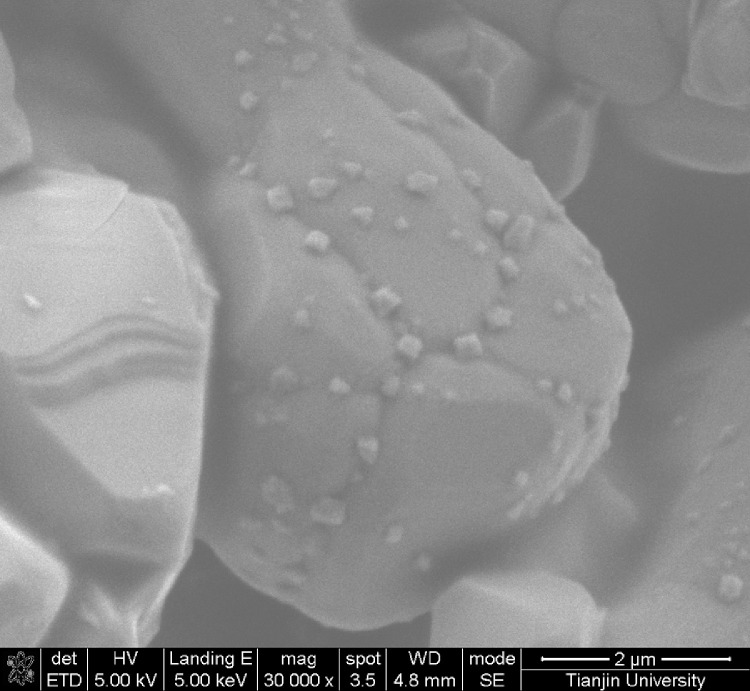
SEM images of morphology of growing whiskers of mixture of Cr2O3:C = 1:8 under conditions of 1500°C - 2 h.

This data article shows the morphologies of products of the mixture of Cr_2_O_3_:C = 1:5 or 1:8 or 1:10 under different temperatures and duration time using SEM or TEM ^[^^1^^]^. More specifically, Fig. 1, Fig. 2 show the morphologies of products of mixture of Cr_2_O_3_:C = 1:5 using SEM under conditions of 1300°C and duration time of 1 h and 4 h, respectively. Some whiskers are shown in both figures. Fig. 3 shows the TEM image of products of a mixture of Cr_2_O_3_:C = 1:5 under conditions of 1300°C and 4 h. Non-whisker products were observed under these experimental conditions. Fig. 4 shows the products of the mixture of Cr_2_O_3_:C = 1:10 under conditions of 1300°C and 1 h using SEM. Whiskers are not obvious in these experimental conditions. Fig. 5 shows the TEM images of products of the mixture of Cr_2_O_3_:C = 1:10 under conditions of 1300°C and 2 h. The agglomerated structures, instead of whiskers, were observed under these experimental conditions. Next, Fig. 6 shows the SEM image of products of the mixture of Cr_2_O_3_:C = 1:10 under conditions of 1300°C and 4 h. Some whiskers can be observed. Finally, Fig. 7 shows the morphology of the products of the mixture of Cr_2_O_3_:C = 1:8 under conditions of 1500°C and 2 h using SEM. Micro-morphology of the growing whiskers can be clearly observed.

## 2 Experimental Design, Materials and Methods

Mechanical performance of ceramics is significant in various applications of engineering structures and their mechanical properties were extensively studied. ^[^[2], [3], [4], [5], [6], [7], [8]^]^ Chromium carbides exhibit superior mechanical properties and chemical stability in harsh environments. ^[^[9], [10], [11], [12]^]^ This work successfully prepared chromium carbide whiskers and showed strengthened mechanical properties for applications in high temperature environment. We chose the polymer-based adhesive ^[^^13^^]^ to be strengthened with whiskers. To prepare the adhesive with whiskers, silicon resin was used as matrix. Silicon, aluminium, boron carbide, and glasses were used as fillers. 7 wt.% whiskers were used as strengthening phases. Furthermore, the original adhesive as well as 3 wt.% short carbon fibers-added adhesive were prepared to be compared. We chose mullite ceramic substrates with sizes of 40 × 10 × 5 smm as adherends and bonded with the above three adhesives into shear test joints, which have the same construction as that from a previous publication.^[^^12^^]^ These joints were tested with CSS-4401 testing machine to compare the strengthening effects after curing and calcination in different temperatures ranging from 500°C to 1500°C. SEM was used to observe the morphology of the adhesives before and after shear test.

X-ray diffractometry (Cu Ka radiation D/Max-2500; Rigaku, Akishima, Japan) was used with a step size of 4°/min between the diffraction angles of 2θ = 20–70° to test the chemical composition of the products.^[^^14^^]^ The morphology comparison, crystal structure, and composition analysis were characterized by SEM^[^^15^^]^ (S-4800; Hitachi,Tokyo, Japan) and field emission gun TEM (Tecnai G2 F20; FEI, Eindhoven, Netherlands) with an energy dispersive X-ray detector (EDS). X-ray photoelectron spectroscopy (XPS, ThermoFisher ESCALAB 250Xi+) was used to identify the elemental species and their respective valences in whiskers. Furthermore, simultaneous thermogravimetric and differential scanning calorimetry (TG-DSC, STA449C, Netzsch Gerätebau, Bavaria, Germany) were used to detect the thermal behaviour of the mixture during sintering and antioxidant property of whiskers in extreme conditions, at a heating rate of 5°C/min from 100°C to 1500°C under a constant argon gas flow of 20 ml/min (the former) and in air (the later).

We put into use the reagents, which are commercially available, without further treatment. Blank carbon (Carter Trade Co., Ltd., Hebei, China), chromic oxide powder (Cr_2_O_3_, 97 wt %, Kmart Chemical Technology Co., Ltd., Tianjin, China), nicker powder (Ni, 99.7 wt %, Bosworth Nanotechnology Co., Ltd., Ningbo, China), and halide salts (NaCl and KCl, 99.9 wt %, Kemiou Chemical Reagent Co., Ltd., Tianjin, China) were adequately blended by high energy ball milling for 24 h. The stoichiometric ratio of Cr_2_O_3:_ C: NaCl: KCl: Ni was 1: 5, 8, 10: 0.4: 0.4: 0.3. The mixtures were dry in an oven at 120°C for 2 h after washed by the distilled water. Then they were grounded in an agate mortar. Finally, the obtained black powders were sintered at different temperatures from 1100°C to 1500°C in high purity argon for various time of 1 h to 4 h.

## Declaration of Competing Interest

The authors declare that they have no known competing financial interests or personal relationships which have, or could be perceived to have, influenced the work reported in this article.
